# Supplementary figures and images for: Classical Mathematical Models for Description and Prediction of Experimental Tumor Growth
Source: PLoS Comput Biol. 2014 Aug 28;10(8):e1003800. doi: 10.1371/journal.pcbi.1003800 (PMC4148196; doi:10.1371/journal.pcbi.1003800)

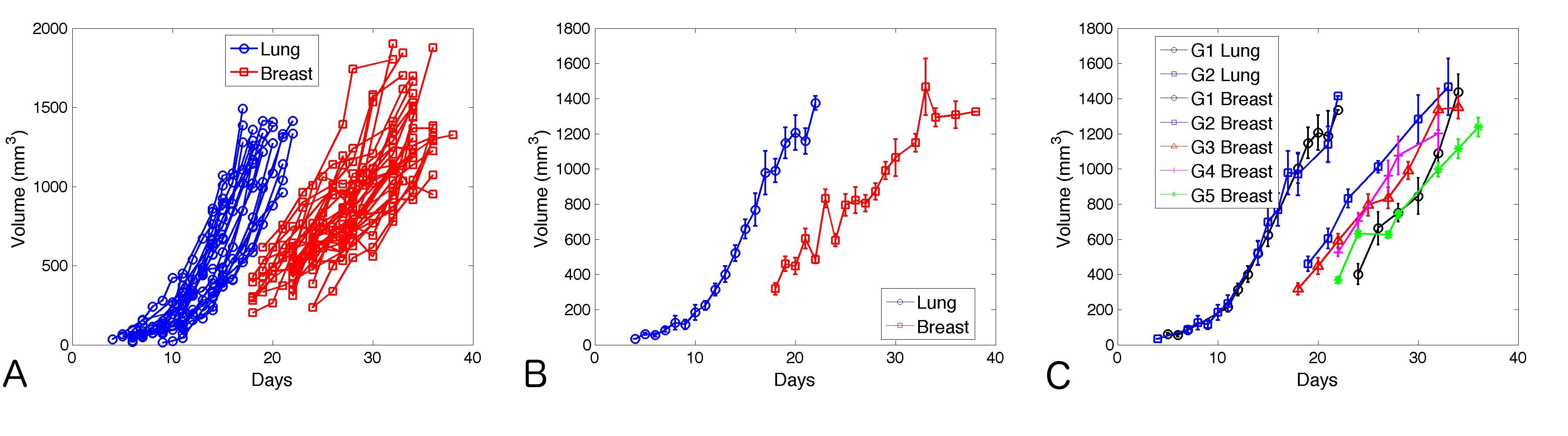

Supplement: Figure S1 — Data. Plots of the brute data sets from the lung and breast experiments. A. All animals' growth curves. B. Average curves. C. Per group averages (lung and breast data resulted from combinations of respectively two and three separate experiments). G = group. (TIF) [file pcbi.1003800.s001.tif]

**Figure S3: Examples of individual predictions. Breast data.**

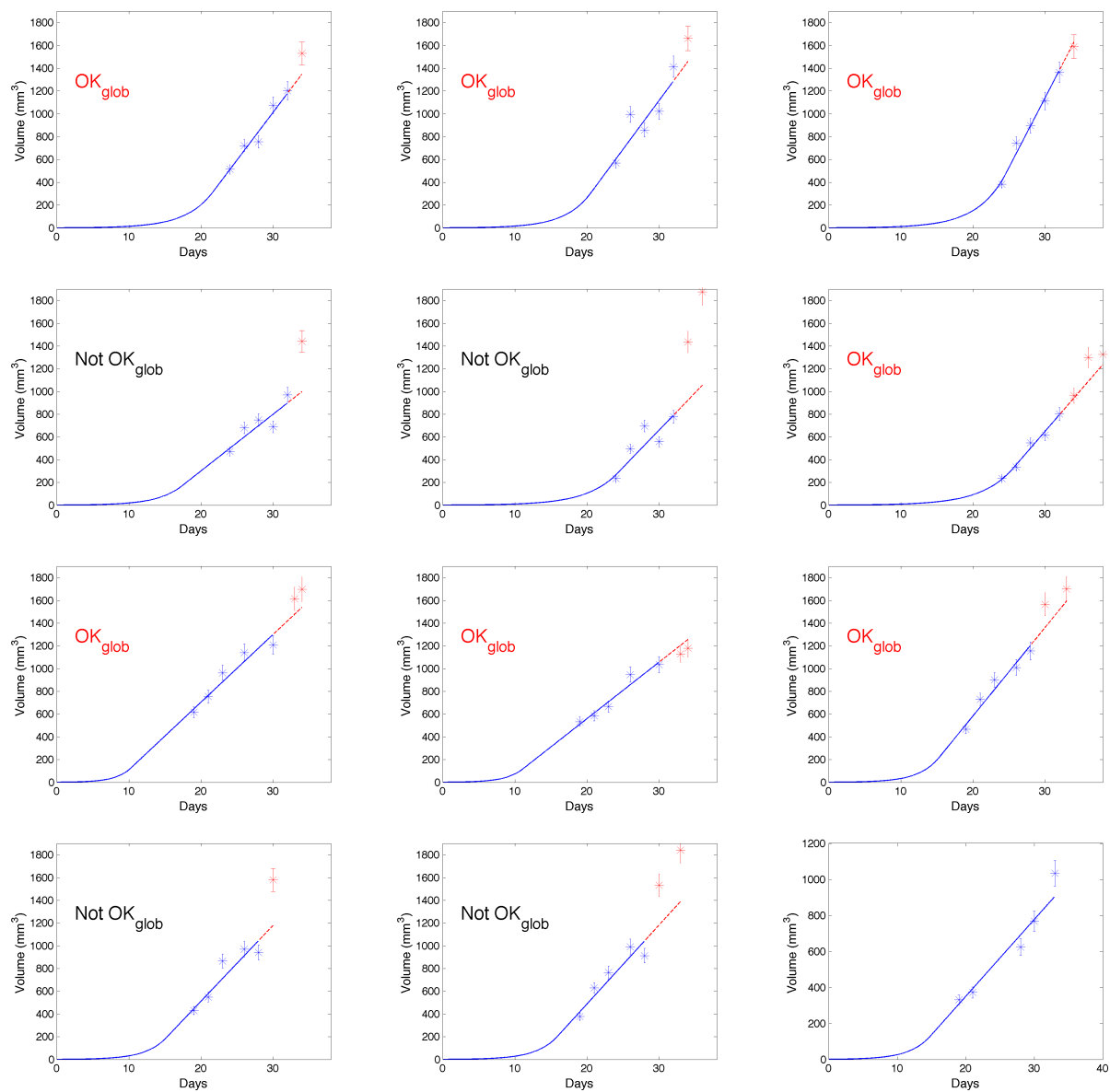

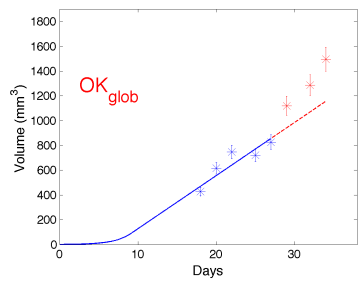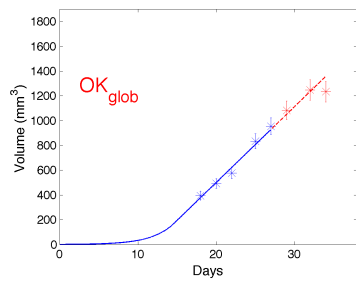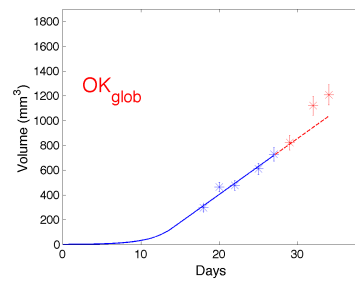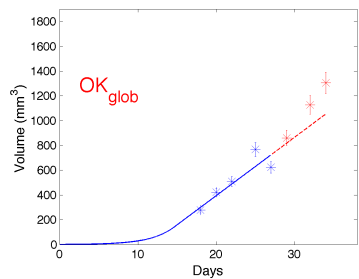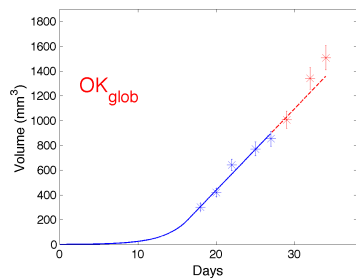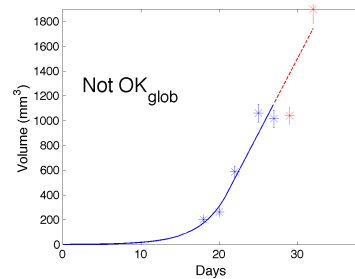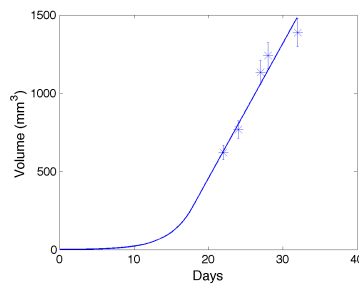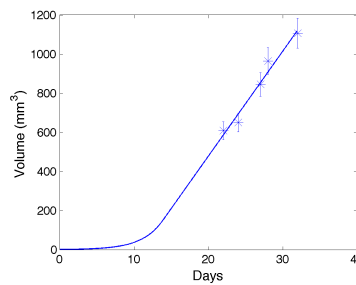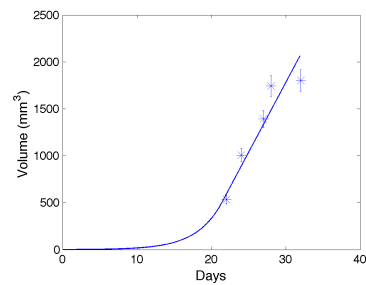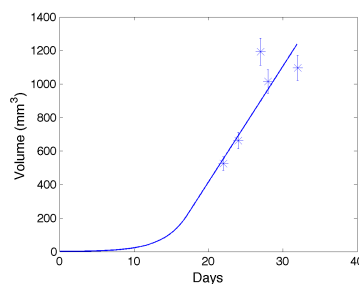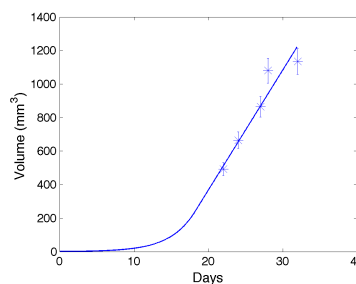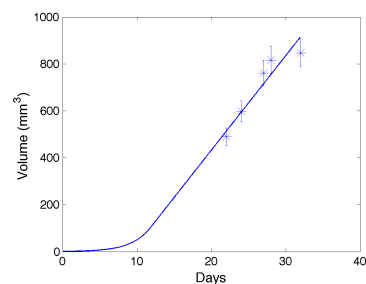

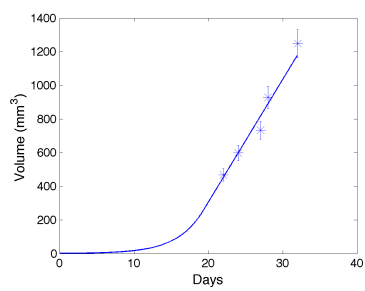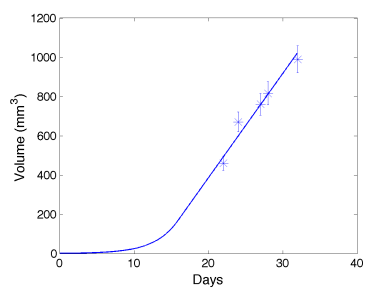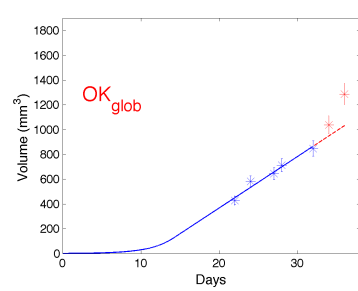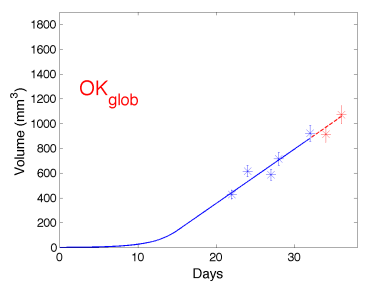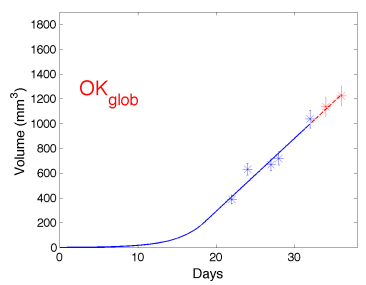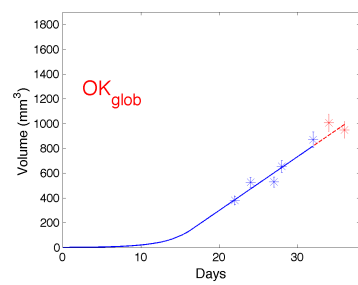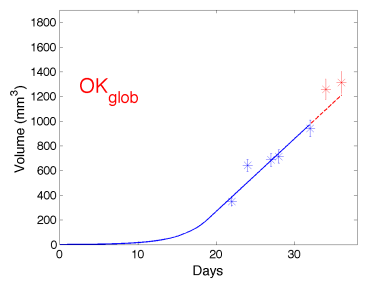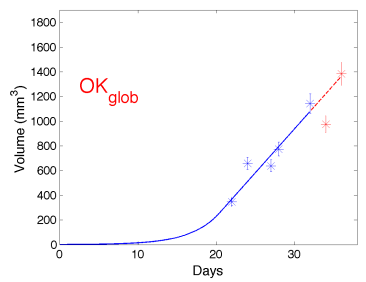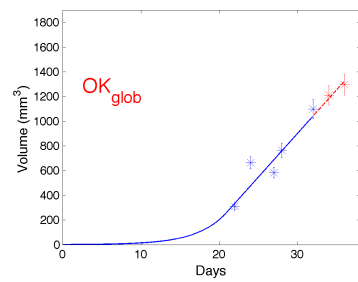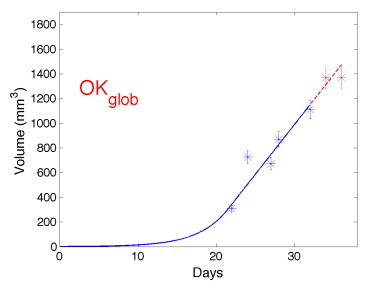

Supplement: Figure S3 — Examples of individual predictions: Breast data. Prediction success of the model are reported for the second next day data point (OK2) or global future curve (OKglob), based on the criterion of a normalized error smaller than 3 (meaning that the median model prediction is within 3 standard deviations of the measurement error) for OK2 and the median of this metric over the future curve for OKglob. Future growth was predicted using 5 data points and the exponential-linear model. (PDF) [file pcbi.1003800.s003.pdf]

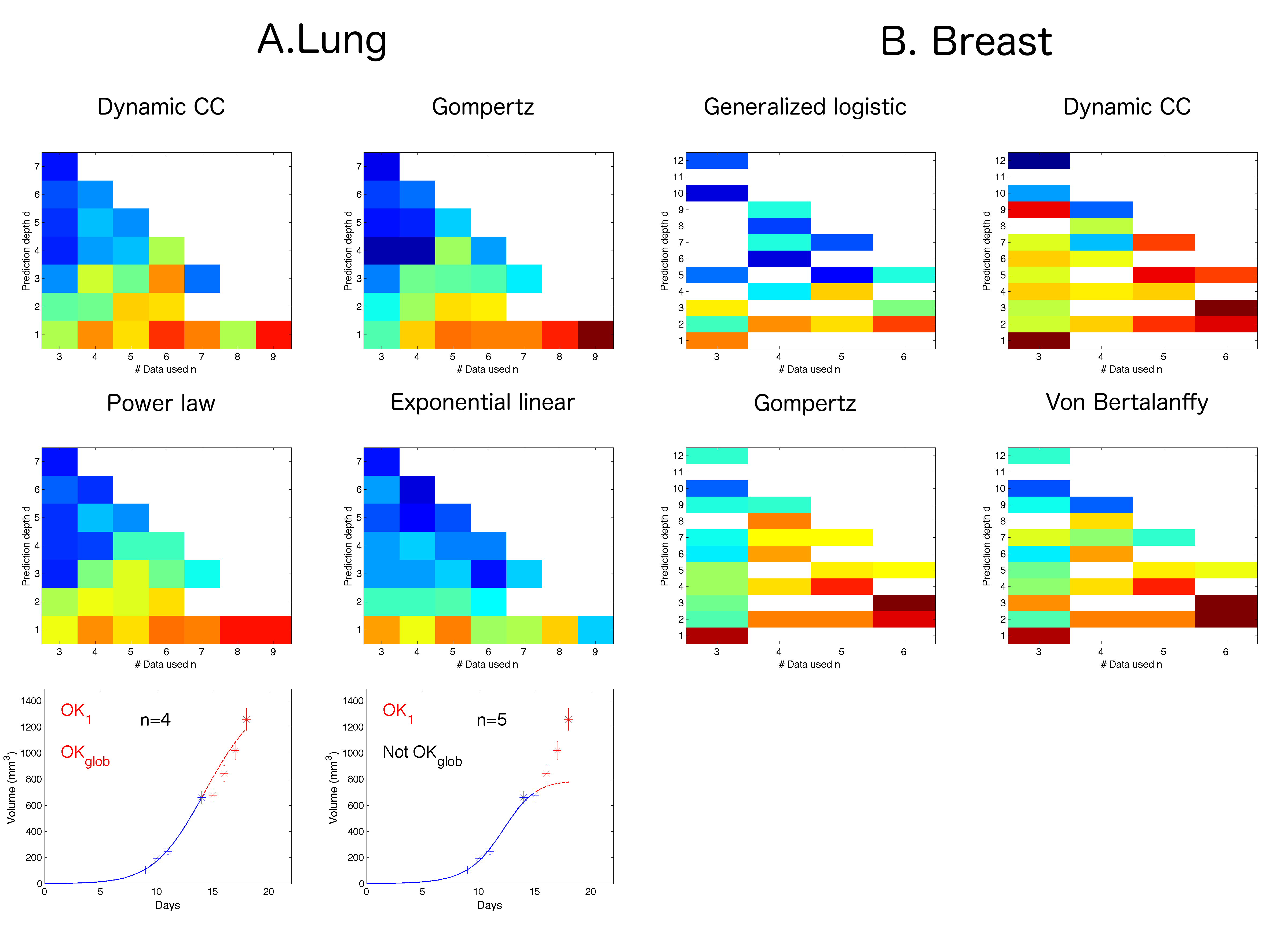

Supplement: Figure S4 — Prediction. Top: Prediction success for models that were not reported in Figure 4. Bottom: Example where prediction was less successful when using n = 5 data points than when using n = 4 data points, with the generalized logistic model. (TIF) [file pcbi.1003800.s004.tif]

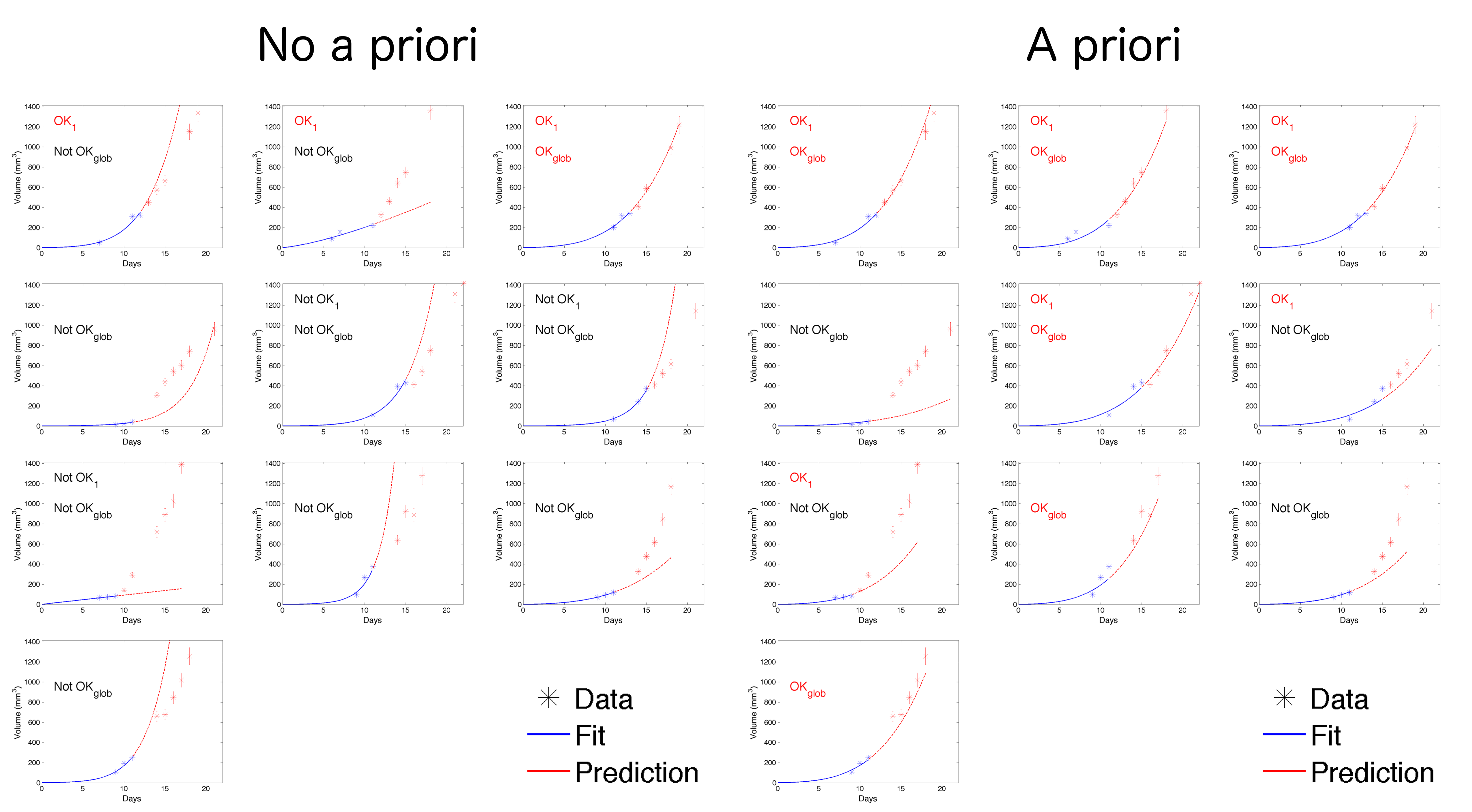

Supplement: Figure S5 — Forecast improvement of the power law model when using a priori information and the lung data set. Fits were performed using the first three data points for each animal. A priori information (learned on a different data set) was added during the fit procedure for the predictions on the right. (TIF) [file pcbi.1003800.s005.tif]
