# Supplementary material for: Classical Mathematical Models for Description and Prediction of Experimental Tumor Growth
Source: PLoS Comput Biol. 2014 Aug 28;10(8):e1003800. doi: 10.1371/journal.pcbi.1003800 (PMC4148196; doi:10.1371/journal.pcbi.1003800)

Figure S2: Examples of individual predictions.

A. Gompertz model (similar to von Bertalanffy, dynamic CC and power law models)

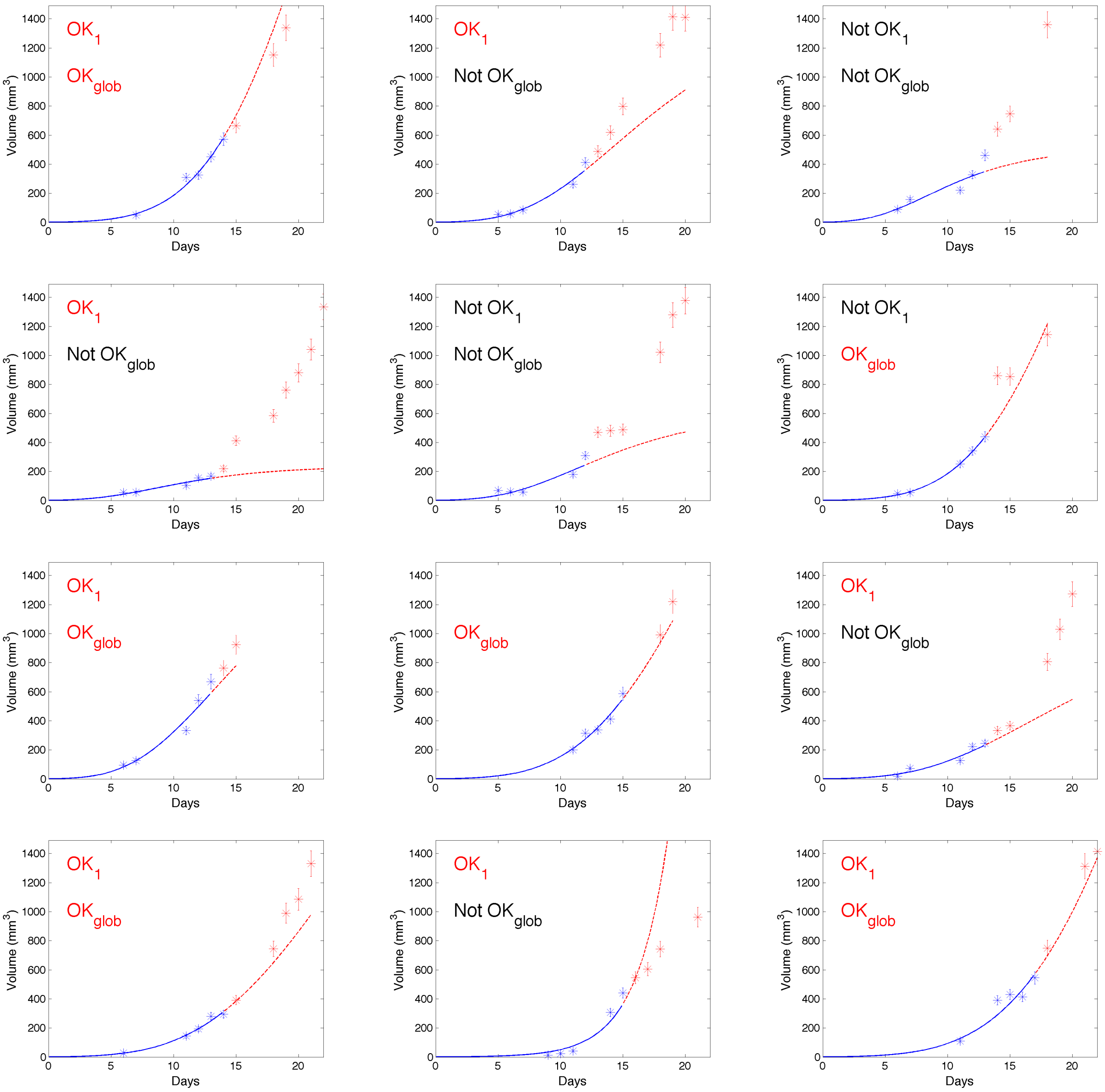

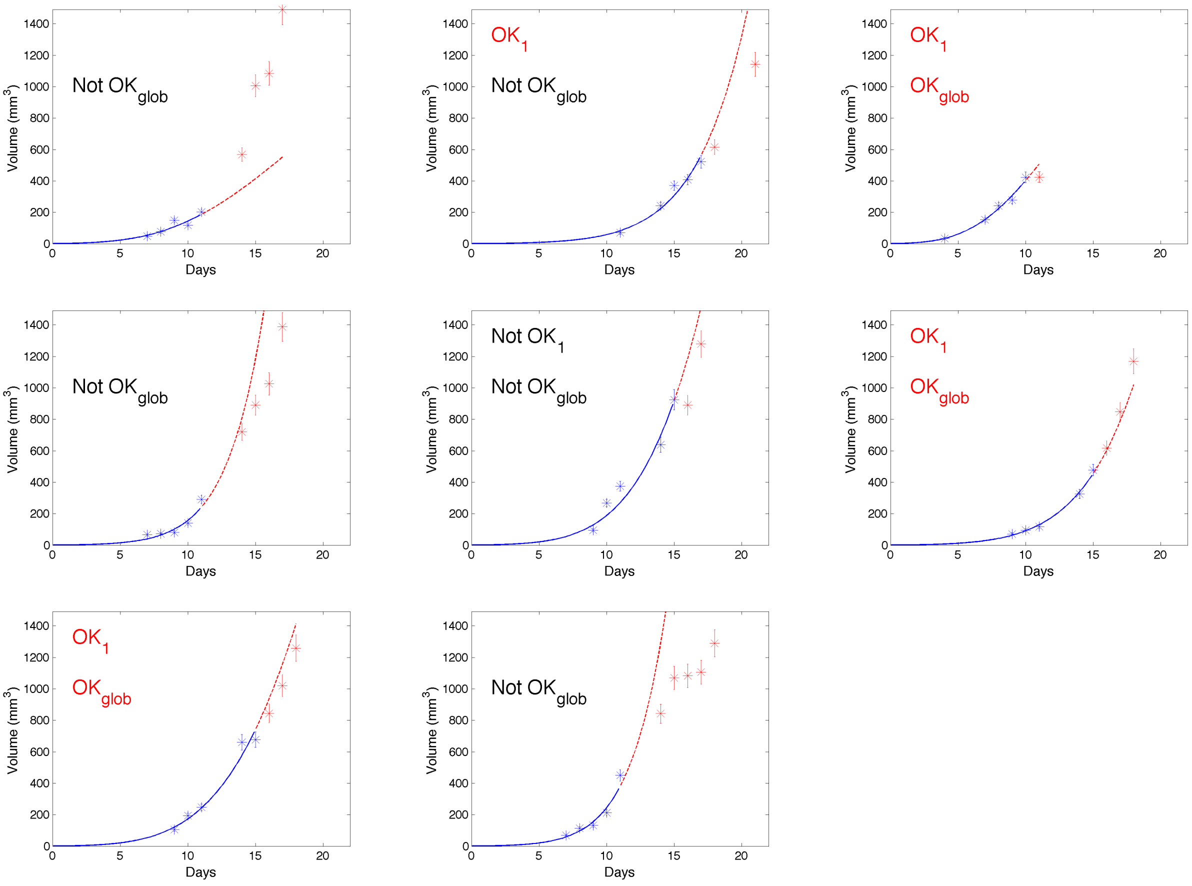

Not OK$_{glob}$
OK$_1$
Not OK$_{glob}$
OK$_1$
OK$_{glob}$
Not OK$_{glob}$
Not OK$_1$
Not OK$_{glob}$
OK$_1$
OK$_{glob}$
OK$_1$
OK$_{glob}$
Not OK$_{glob}$
Volume (mm^3)
Days
0
200
400
600
800
1000
1200
1400
0
5
10
15
20

B. Examples of sharp saturation of the generalized logistic model

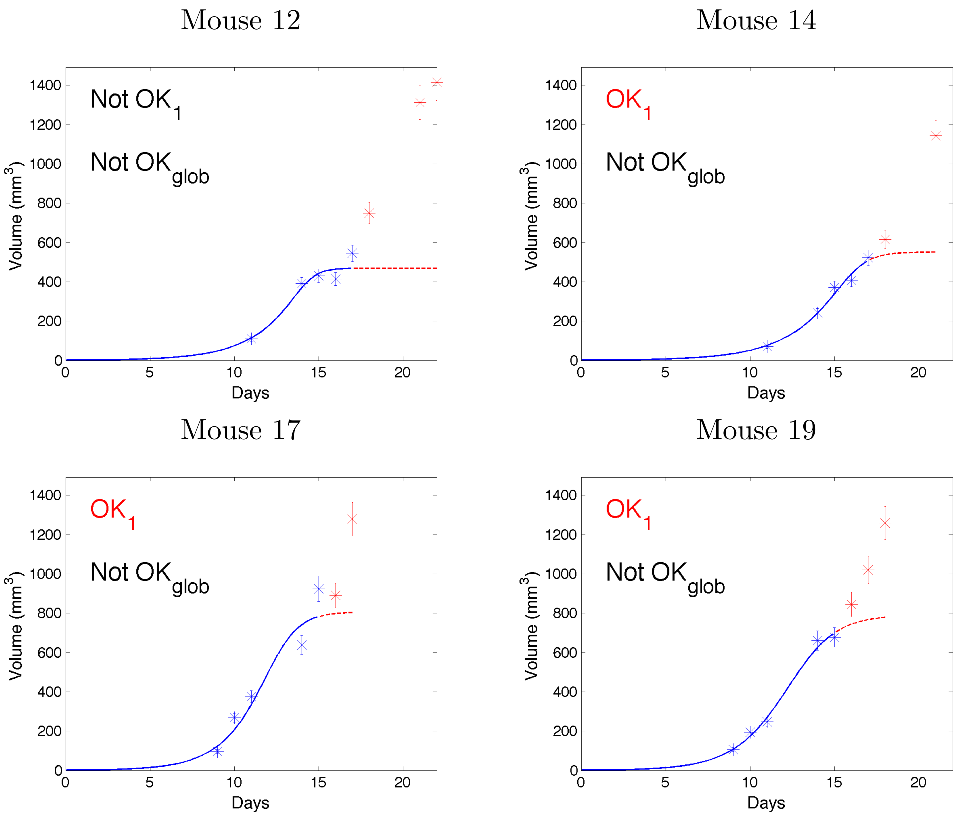

Supplement: Figure S2 — Examples of individual predictions: Lung data. Prediction success of the model are reported for the next day (OK1) or global future curve (OKglob), based on the criterion of a normalized error smaller than 3 (meaning that the median model prediction is within 3 standard deviations of the measurement error) for OK1 and the median of this metric over the future curve for OKglob. Future growth was predicted using 5 data points and the von Bertalanffy model. (PDF) [file pcbi.1003800.s002.pdf]
