# Supplementary material for: Classical Mathematical Models for Description and Prediction of Experimental Tumor Growth
Source: PLoS Comput Biol. 2014 Aug 28;10(8):e1003800. doi: 10.1371/journal.pcbi.1003800 (PMC4148196; doi:10.1371/journal.pcbi.1003800)
Supplement: Table S1 — Initializations of the least squares minimization algorithm. Also reported are bounds used in lsqcurvefit for estimation of parameters of the power law, von Bertalanffy and exponential-linear models. (DOCX) [file pcbi.1003800.s006.docx]

**Table S1: Initializations of the least squares minimization algorithm.**

| **Model** | **Parameter** | **Initialization** | **Upper bound** |
| --- | --- | --- | --- |
| Power law | $a$ | 1 | 100 |
|  | $\gamma$ | 2/3 | 1 |
| Gompertz | $a$ | 1 | - |
|  | $\beta$ | 0.1 | - |
| Dynamic CC | $a$ | 3 | - |
|  | $b$ | 0.5 | - |
|  | $K_{0}$ | 10 | - |
| Generalized logistic | $a$ | 10 | - |
|  | $K$ | 10000 | - |
|  | $\alpha$ | 0.01 | - |
| Von Bertalanffy | $a$ | 1 | 100 |
|  | $\gamma$ | 0.75 | 1 |
|  | $b$ | 0.1 | 100 |
| Exponential $V_{0}$ | $a$ | 0.1 | - |
|  | $V_{0}$ | 20 | - |
| Exponential-linear | $a_{0}$ | 0.2 | 10 |
|  | $a_{1}$ | 100 | 200 |
| Logistic | $a$ | 1 | - |
|  | $K$ | 10000 | - |
| Exponential 1 | $a$ | 0.1 | - |

Also reported are bounds used in *lsqcurvefit* for estimation of parameters of the power law, von Bertalanffy and exponential-linear models.
