# Supplementary material for: Classical Mathematical Models for Description and Prediction of Experimental Tumor Growth
Source: PLoS Comput Biol. 2014 Aug 28;10(8):e1003800. doi: 10.1371/journal.pcbi.1003800 (PMC4148196; doi:10.1371/journal.pcbi.1003800)
Supplement: Table S2 — Comparison of individual fits between the individual and population approaches. Fits were performed using either an individual estimation of the growth curves based on weighted least-squares estimation or a population approach. In both settings, the error model was proportional to the volume to the power α = 0.84. The only difference was that Monolix estimation did not allow for a setting with a threshold volume Vm, which was thus taken to be 0. However, due to its low value (Vm = 83 mm3), it was not very active in the individual approach. Reported are the mean (over the time points) weighted least squares (i.e. the ones of the first column of Tables 1, 2 except with Vm = 0, i.e. the Monolix setting), for both approaches. More precisely, if with being the individual estimate of parameter set β in animal j, using either the individual approach () or the population approach (). The numbers reported are the mean value of (over the population, i.e. over index j) as well as minimal and maximal values. Note that due to the relatively large volumes of the breast data, Vm was not active and the values of the individual approach are exactly the ones of Table 2 in this case (S2.B). Last column is the p-value of Student's t-test for significant differences between the individual and population approaches. A. Lung data set. B. Breast data set. (PDF) [file pcbi.1003800.s007.pdf]

Tables S2: Comparison of individual fits between the individual and population approaches.

S2.A Lung data

Model	Indiv. (least squares)	Pop. (Monolix)	p
DynCC	0.236(0.0143-2.33)	0.515(0.0228-6.66)	0.421
Gomp	0.274(0.0213-2.74)	0.768(0.0422-10)	0.335
PL	0.27(0.0176-2.68)	0.988(0.0362-13.9)	0.308
VonBert	0.256(0.0176-2.74)	0.735(0.032-10.5)	0.374
ExpV0	0.373(0.00718-2.9)	0.947(0.0638-12.4)	0.36
GLog	0.186(0.0213-1.25)	0.799(0.0478-10.3)	0.234
Log	0.349(0.0547-1.81)	0.977(0.12-4.38)	0.0471
Exp-L	0.322(0.0529-1.32)	0.614(0.0621-3)	0.12
Exp1	1.46(0.335-2.58)	2.53(0.924-10.7)	0.0686

S2.B Breast data

**Model**	**Indiv. (least squares)**	**Pop. (Monolix)**	p
DynCC	0.11(0.018-0.503)	0.25(0.0352-1.75)	0.0448
Gomp	0.0976(0.0147-0.328)	0.237(0.039-1.87)	0.0306
PL	0.102(0.0159-0.323)	0.216(0.0311-1.49)	0.0367
VonBert	0.0928(0.0148-0.323)	0.263(0.0337-2.11)	0.0231
ExpV0	0.118(0.0106-0.37)	0.278(0.0121-2.17)	0.0484
GLog	0.0814(0.00366-0.328)	0.226(0.0361-1.8)	0.0181
Log	0.145(0.00367-0.417)	0.178(0.0234-0.66)	0.232
Exp-L	0.0919(0.0159-0.49)	0.113(0.0273-0.615)	0.394
Exp1	2.19(0.617-3.44)	5.88(0.629-27.4)	0.00157
