# Supplementary material for: Classical Mathematical Models for Description and Prediction of Experimental Tumor Growth
Source: PLoS Comput Biol. 2014 Aug 28;10(8):e1003800. doi: 10.1371/journal.pcbi.1003800 (PMC4148196; doi:10.1371/journal.pcbi.1003800)
Supplement: Table S3 — Practical identifiability. Two identifiability scores were reported. The fit score is the proportion of minimization runs, among the 20× NP performed, for which the resulting minimized objective converged to the same value as when starting from the baseline value. The global parametric score is the proportion of minimization runs that converged to the same parameter vector, within a 10% relative error. When this last score was lower than 100%, further analysis was conducted and the same score was computed for each parameter of the model. We also reported their median relative deviation to the base value, in percent. Par. = Parameter. Dev. = Deviation. (PDF) [file pcbi.1003800.s008.pdf]

Table S3: Practical identifiability. Two identifiability scores were reported.The fit score is the proportion of minimization runs, among the $20 \times N^P$ performed,for which the resulting minimized objective converged to the same valueas when starting from the baseline value. The global parametric score is the proportion of minimization runs that converged to the same parameter vector, within a 10% relative error. When this last score was lower than 100%, further analysis was conducted and the same score was computed for each parameter of the model. We also reported their median relative deviation to the base value, in percent. Par. = Parameter. Dev. = Deviation.

**Model**	**Fit score (%)**	**Global par. score (%)**	**Par. score (%) (Median Dev.)**		
Power law	100.0	100.0	a: 100.0 (0.000811)	γ: 100.0 (0.000272)	
Gompertz	100.0	100.0	a: 100.0 (0.000273)	β: 100.0 (0.000662)	
Dynamic CC	88.6	44.5	a: 79.1 (0.71)	b: 90.2 (0.357)	K_0: 45.2 (19.3)
Von Bertalanffy	99.4	34.4	a: 77.9 (1.53)	γ: 99.3 (0.0181)	b: 34.4 (21.3)
Generalized logistic	93.6	56.5	a: 56.9 (4.92)	K: 93.4 (0.00149)	ν: 57.0 (5.01)
Exponential V_0	100.0	100.0	V_0: 100.0 (0.00134)	a: 100.0 (0.000366)	
Logistic	97.7	97.7	a: 97.7 (0.001)	K: 97.7 (0.00557)	
Exponential-linear	95.0	97.3	a_0: 97.3 (0.000517)	a_1: 97.3 (0.00262)	
Exponential 1	100.0	100.0	a: 100.0 (0.00013)		
