# Supplementary material for: Classical Mathematical Models for Description and Prediction of Experimental Tumor Growth
Source: PLoS Comput Biol. 2014 Aug 28;10(8):e1003800. doi: 10.1371/journal.pcbi.1003800 (PMC4148196; doi:10.1371/journal.pcbi.1003800)
Supplement: Text S1 — Numerical procedures for estimation of parameters. (DOCX) [file pcbi.1003800.s009.docx]

**Text S1: Numerical procedures for parameters estimation**

When available, we used the analytical formula of the model for numerical computations. This was the case for all the models except the dynamic CC model (5), for which we used the ode solver *ode45* of Matlab for computation of the solution.

Several optimization algorithms implemented in Matlab [49] were preliminary tested for minimization of $\chi^{2}$ and the function *lsqcurvefit* (with trust-region algorithm and maximum number of iterations and function evaluations allowed both set to ${10}^{5}$) was decided to be well-suited for most of the settings based on its rapidity of convergence and ability to manage bounds constraints. Convergence of the algorithm was systematically checked and was effective for all the parameter estimations performed (for both the descriptive and predictive analysis). Values of the parameters used for initialization of the minimization algorithm were fixed from preliminary explorative analysis, sometimes with the help of the more global optimizer *fminsearch*. They are reported in the Table S1 and a short study of the impact of the initial guess on the values of the parameters resulting from the optimization is provided in the supplementary text S2 and supplementary Table S3.

We compared the fits obtained by *lsqcurvefit* to other regression functions implemented in Matlab, namely *nlinfit* (Levenberg-Marquardt algorithm) or *fminsearch* (Nelder-Mead algorithm). For the identifiable models (in the sense of the practical identifiability reported in text S2), as well as for the dynamic CC model, no significant differences in the estimated parameter sets were observed (Student’s t-test) when comparing fits obtained with either *nlinfit* or *fminsearch* to the fits of *lsqcurvefit*. For instance, the relative difference for parameters estimates of the full lung data set with the power law model was lower than $1.5\times{10}^{-4}$ and $p$ values of Student’s t-test for significant differences between the population distributions of parameter sets were all larger than 0.99. On the other hand, consistently with their dependence on the initialization of the optimization algorithm (Table S2), parameters estimations of the two non-identifiable models generalized logistic (3) and von Bertalanffy (6), were significantly sensitive to the algorithm used. However, for the von Bertalanffy model, this difference in the parameters did not impact on the resulting growth curve and generated the same fits (identical value of the minimal sum of squared errors). This observation suggests a structural non-identifiability of this model for the range of the observed dynamics, that is, that different parameter values can generate the same curve.

Two models required specific attention, namely the generalized logistic model (3) and the exponential-linear model (1). In the first setting, due to low identifiability, *lsqcurvefit* was found to converge to irrealistic local minima and we rather used the more global optimizer *fminsearch* with an increased maximum number of iterations and function evaluations allowed (from 600 to 10^5^), in order to ensure convergence of the algorithm. Convergence was checked *a posteriori* to be effective*.* For the exponential-linear model, local minima issues were also likely to happen because, on a finite time-course and within some parameter ranges, the model could be insensitive to variation of one of the parameter and remain in a fully-linear or fully-exponential phase (if the resulting $\tau$ was larger than the maximum time or lower than the minimal one), which could be sub-optimal. A useful workaround was to impose upper bounds on the parameters (in particular $a_{1}$). To avoid numerical issues when solving the von Bertalanffy and power law models, we also imposed an upper bound on parameter $\gamma$. In the setting of *lsqcurvefit*, this required imposition of a bound also on parameters $a$ and $b$, which were taken large enough not to be active.
