# Supplementary material for: Classical Mathematical Models for Description and Prediction of Experimental Tumor Growth
Source: PLoS Comput Biol. 2014 Aug 28;10(8):e1003800. doi: 10.1371/journal.pcbi.1003800 (PMC4148196; doi:10.1371/journal.pcbi.1003800)
Supplement: Text S2 — Practical identifiability of the models. (DOCX) [file pcbi.1003800.s010.docx]

**Text S2: Practical identifiability of the models**

Sensitivity of the fits obtained from our estimation procedure was assessed for each model by systematically varying initialization of the algorithm. Only the LLC tumor data set was used for this study, in order to limit the computational cost and because the results should be similar with the LM2-4^LUC+^ data. A compact subset of the parameter space of length 4 standard deviations was meshed and explored in each parameter direction above and below the mean population value from the fits reported in the text (Table 3). For the generalized logistic and von Bertalanffy models, due to high inter-animal variability of the parameters estimates (Table 3), this method led to extreme values of the parameters. These were not relevant initializations because they resulted in extreme, biologically unrealistic, behaviors of the models. Starting from these initializations, the minimization procedure was unable to generate good fits and consequently the identifiability scores were very low, but for a methodological reason and not due to structural properties of the models. Consequently, a different procedure was employed. The baseline mean value was taken to be the one of Table S1 and standard deviation was replaced by an arbitrary standard deviation of 50% of the baseline value. For each model, $20\times{11}^{P}$ individual fits were performed (for the 20 animals growth curves) with $P$ the number of parameters in the model, giving a total of 92180 individual tumor growth performed for the analysis.

We reported in Table S3 results of sensitivity scores. The first score was defined as the fraction of minimization runs (over all the animals and initializations) for which the minimized objective (defined in (8)) converged to the same value as when starting from the baseline initialization (within a 10% relative error). This score essentially verifies that all the resulting fits, even if converging to a different parameter vector than with the baseline initialization, were equally good. A low fit score would mean that the optimization algorithm has often converged to sub-optimal minima. All of the models had very good fit score (Table S3).

The second score concerned parametric identifiability and was defined as the fraction of minimization runs that converged to the same parameter vector as when performing minimization starting from the baseline value (within a 10% relative error). When global numerical identifiability was not observed, further investigation on each parameter was performed and the resulting variation of the best-fit parameter computed (Table S3). Low parametric identifiability scores means that the model was able to generate almost identical growth curves with different parameter sets.

As could be expected from their large standard errors on the parameters estimates (Table 3), the models with three parameters (von Bertalanffy (6), dynamic CC (5) and generalized logistic (3)) exhibited low parametric identifiability scores (of respectively 34.4%, 45.1% and 56.5%, out of 26620 fits for each). For the von Bertalanffy model, the non-identifiability was probably due to the absence of identifiable range of convergence to an asymptotic volume in our data. The low identifiability of the dynamic CC model can be explained by its two-dimensional nature (volume and carrying capacity are variables) with only one observable used for the fits. This fact resulted in variability mostly in estimation of $K_{0}$. For the generalized logistic model, non-identifiability mostly came from parameters $a$ and $\nu$. This could be explained by the high flexibility of the model that generated several close-to-optimal fits, although with different parameter values. Most of the fits (93.6%) were in the range of 10% from the baseline fit (i.e. the fits used in Table 1). Considering a different parameterization of the model (changing $a$ into $\tilde{a}=\frac{a}{\nu}$) might improve the identifiability of the generalized logistic model.

All the other models exhibited very good robustness in the parameter estimation.
